# Methodological standards for body composition—an expert-endorsed guide for research and clinical applications: levels, models, and terminology

**DOI:** 10.1016/j.ajcnut.2025.05.022

**Published:** 2025-07-17

**Authors:** Carla M Prado, M Cristina Gonzalez, Kristina Norman, Rocco Barazzoni, Tommy Cederholm, Charlene Compher, Gordon L Jensen, Takashi Abe, Thiago Gonzalez Barbosa-Silva, Anja Bosy-Westphal, Owen T Carmichael, Carrie P Earthman, William J Evans, David A Fields, Laurence Genton, Houchun Harry Hu, Murat Kara, Jennifer L Miles-Chan, Marina Mourtzakis, Manfred J Muller, Camila E Orsso, Stany Perkisas, Luís B Sardinha, John A Shepherd, Mario Siervo, Boyd JG Strauss, Yosuke Yamada, Shankuan Zhu, Steven B Heymsfield

**Affiliations:** 1 Department of Agricultural, Food and Nutritional Science, Faculty of Agricultural, Life and Environmental Sciences, University of Alberta, Edmonton, Alberta, Canada; 2 Postgraduate Program in Nutrition and Food, Federal University of Pelotas, Pelotas, Rio Grande do Sul, Brazil; 3 Department of Geriatrics, Charité Universitätsmedizin Berlin, Berlin, Germany; 4 Department of Nutrition and Gerontology, German Institute of Human Nutrition Potsdam-Rebrücke, Nuthetal, Germany; 5 Department of Medical, Surgical and Health Sciences, University of Trieste, Ospedale di Cattinara, Trieste, Italy; 6 Department of Public Health and Caring Sciences, Clinical Nutrition and Metabolism, Uppsala University, Uppsala, Sweden; 7 Theme Inflammation and Ageing, Karolinska University Hospital, Stockholm, Sweden; 8 Department of Biobehavioral Health Science, University of Pennsylvania, School of Nursing, Philadelphia, PA, United States; 9 Dean’s Office and Department of Medicine, University of Vermont Larner College of Medicine, Burlington, VT, United States; 10 Institute of Health and Sports Science & Medicine, Graduate School of Health and Sports Science, Juntendo University, Chiba, Japan; 11 Department of General Surgery, Faculty of Medicine, Federal University of Pelotas, Pelotas, Rio Grande do Sul, Brazil; 12 Institute of Human Nutrition and Food Science, Faculty of Agricultural and Nutritional Sciences, Christian-Albrechts-University, Kiel, Germany; 13 Pennington Biomedical Research Center, Louisiana State University System, Baton Rouge, LA, United States; 14 Department of Health Behavior and Nutrition Sciences, College of Health Sciences, University of Delaware, Newark, DE, United States; 15 Department of Nutritional Sciences and Toxicology, University of California Berkeley, Berkeley, California, United States; 16 Division of Geriatrics, Duke University School of Medicine, Durham, NC, United States; 17 Department of Pediatrics, Section of Diabetes and Endocrinology, University of Oklahoma Health Sciences Center, Oklahoma City, OK United States; 18 Clinical Nutrition, Department of Medicine, Geneva University Hospitals, Geneva, Switzerland; 19 Department of Radiology, University of Colorado, Anschutz School of Medicine, Aurora, CO, United States; 20 Department of Physical Medicine and Rehabilitation, Hacettepe University Medical School, Ankara, Turkey; 21 Human Nutrition Unit, School of Biological Sciences, University of Auckland, Auckland, New Zealand; 22 Department of Kinesiology and Health Sciences, Faculty of Health, University of Waterloo, Waterloo, Canada; 23 University Center for Geriatrics, University of Antwerp/ZNA, Antwerp, Belgium; 24 Exercise and Health Laboratory, CIPER, Faculdade de Motricidade Humana, Universidade de Lisboa, Cruz Quebrada, Portugal; 25 Department of Epidemiology, University of Hawaii Cancer Center, Honolulu, HI, United States; 26 School of Population Health, Curtin University, Perth, Western Australia, Australia; 27 Curtin Dementia Centre of Excellence, enAble Institute, Curtin University, Perth, WA, Australia; 28 Department of Medicine, School of Clinical Sciences, Monash University, Melbourne, VIC, Australia; 29 Division of Diabetes, Endocrinology and Gastroenterology, School of Medical Sciences, Faculty of Biology, Medicine and Health, The University of Manchester, Manchester, United Kingdom; 30 Graduate School of Biomedical Engineering, Tohoku University, Sendai, Japan; 31 Department of Medicine and Science in Sports and Exercise, Graduate School of Medicine, Tohoku University, Sendai, Japan; 32 Chronic Disease Research Institute, The Children's Hospital, and National Clinical Research Center for Child Health, School of Public Health, School of Medicine, Zhejiang University, Hangzhou, China; 33 Department of Nutrition and Food Hygiene, School of Public Health, School of Medicine, Zhejiang University, Hangzhou, China

**Keywords:** body composition, definitions, terminology, adipose tissue fat-free mass, fat mass, lean mass, lean soft tissue, skeletal muscle

## Abstract

Body composition assessment is widely used in both research and clinical practice, yet confusion over basic concepts and terminology persists, leading to inaccurate assessments, comparisons, and interpretations. To address this concern, an international working group was formed to clarify basic concepts, standardize terminology, and provide guidance on the use and interpretation of body composition assessment. This initial publication addresses methodological standards, focusing on summarizing body composition levels and models, and introducing standardized terms and definitions. Body composition is organized into 5 distinct levels, ranging from atomic to whole-body, with each higher level encompassing the components of the preceding less complex levels. As a result, terms that describe components at different levels should not be used interchangeably. For example, the use of the molecular-level term “lean body mass” is discouraged because it inaccurately refers to fat-free mass (FFM), lean mass, or lean soft tissue (LST). FFM includes all compartments at the molecular level except fat (nonpolar lipids; mainly triglycerides), and FFM also contains nonfat (or polar) lipids. The term “lean mass” is equivalent to FFM, but not to LST, as FFM includes bone mineral content. Additionally, skeletal muscle is classified at the tissue-organ level and should not be confused with the molecular-level components FFM and LST. Likewise, fat mass and adipose tissue are different components: fat mass, mainly triglycerides, is assessed at the molecular level, whereas adipose tissue is measured at the tissue-organ level. Models are also specific to each level. It is crucial for researchers and clinicians to have a clear understanding of what each body component entails and to use accurate terminology to ensure precise assessment, reporting, and interpretation of body composition data.

## Introduction

The study of human body composition has emerged as a major component of research and clinical practice in medicine, nutrition, and exercise science fields. An online literature search performed in PubMed on 24 April, 2025, using the search string (human) AND (body composition) identified over 98,000 publications reporting “human body composition” at the time of this publication. Over the past 10 y, these have been published at a rate of over 4700 per y, with the first appearing in 1842 [1], followed by a large gap up until the 1950s (Supplemental Figure 1). As such, thousands of scientific and lay publications, guidelines/reports, conference presentations, and educational resources have addressed the relationships between body composition and health outcomes. Nevertheless, there is often a misunderstanding of basic concepts and terminology, negatively impacting data presentation, reporting, interpretation, and comparability of findings.

These recent developments led us to create an international working group comprising 29 body composition experts to clarify concepts, standardize the scientific terminology used in reporting body composition research, and provide practical advice for the use and interpretation of body composition in both research and clinical settings. An advisory group with clinical expertise (RB, TC, CC, GLJ) ensured the appropriateness of the developed messages to clinicians and alignment to relevant clinical guidelines. Our a priori approach was consensus among working group members, whereby any disagreements in the final manuscript version would be disclosed and discussed.

This methodological standard is the first publication of a planned series. It was concisely written, primarily for those who use body composition methods but may not be experts in their application. This article is presented in a manner that facilitates its use and dissemination across various disciplines, reflecting the current state of the scientific literature. In this initial publication, we summarize body composition levels and models and present standardized terminologies and definitions. Upcoming publications will report on principles of common methods. Notably, our methodological standard series is focused specifically on adult body composition. Pediatric body composition, which differs in several aspects, is beyond the scope of this work and would be more appropriately addressed in a separate standard dedicated to that population. In fact, there is a clear need in the pediatric literature for an initiative to establish methodological standards for assessing, interpreting, and reporting body composition in children and youth.

## Overview of Body Composition Levels and Models

**FIGURE 1:**
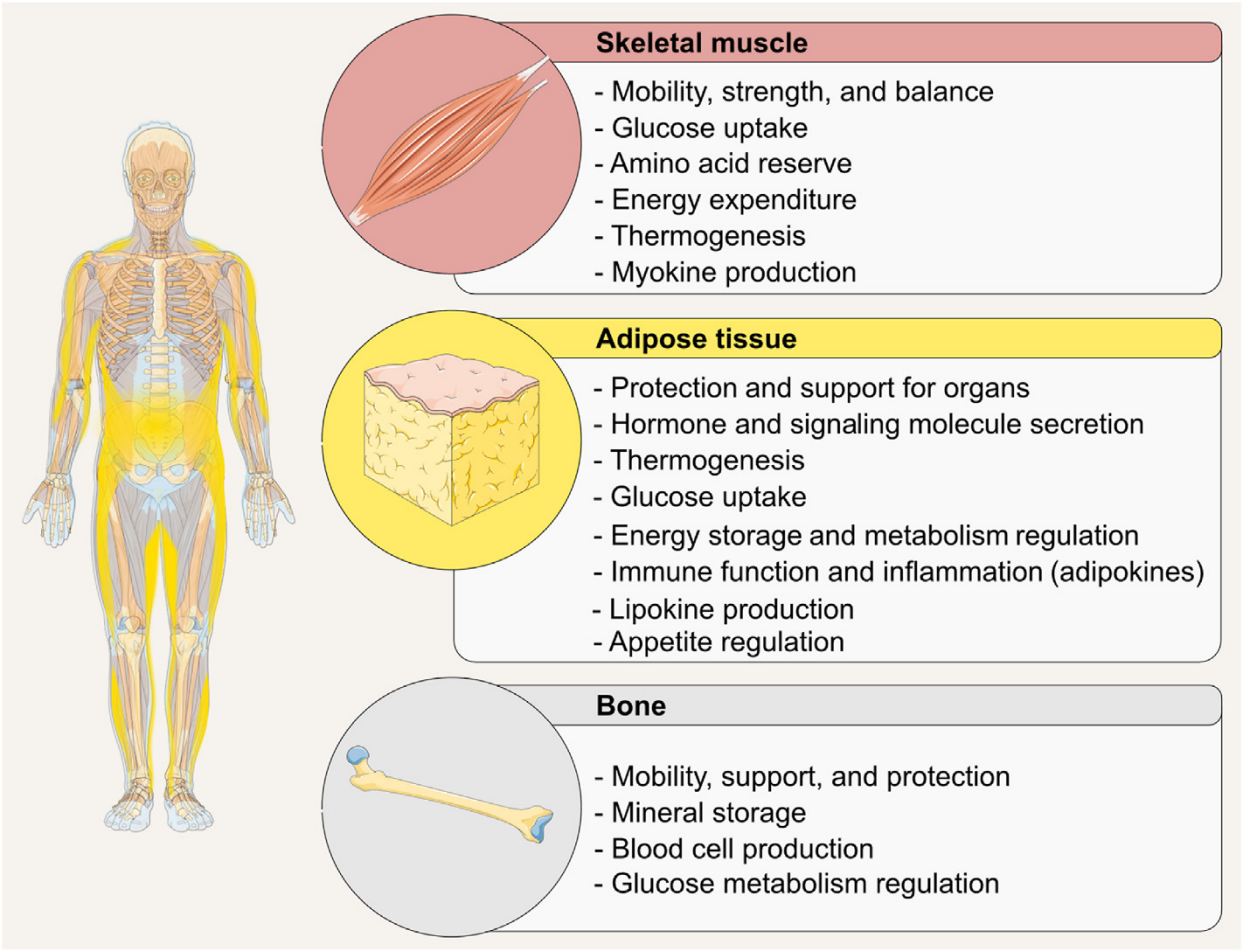
Selected functions of skeletal muscle, adipose tissue, and bone. Images retrieved from smart.servier.com.

Body composition is a direct reflection of the net lifetime accumulation and losses of macronutrients, water, minerals, and other substrates. From simple elements to complex tissues and organs, components retained by the body serve as essential building blocks that confer mass, shape, and function to all living beings (Figure 1) [2]. To better study each body component, body composition has been organized into different levels of complexity. A core framework proposed by Wang et al. [3] divides the body into 5 levels of increasing complexity and provides a structured approach for quantifying various components of the body, aligning with the different levels of biological organization. The 5-level organizational framework was later validated [4,5], and these levels form the foundational basis for body composition assessment methods.

**FIGURE 2:**
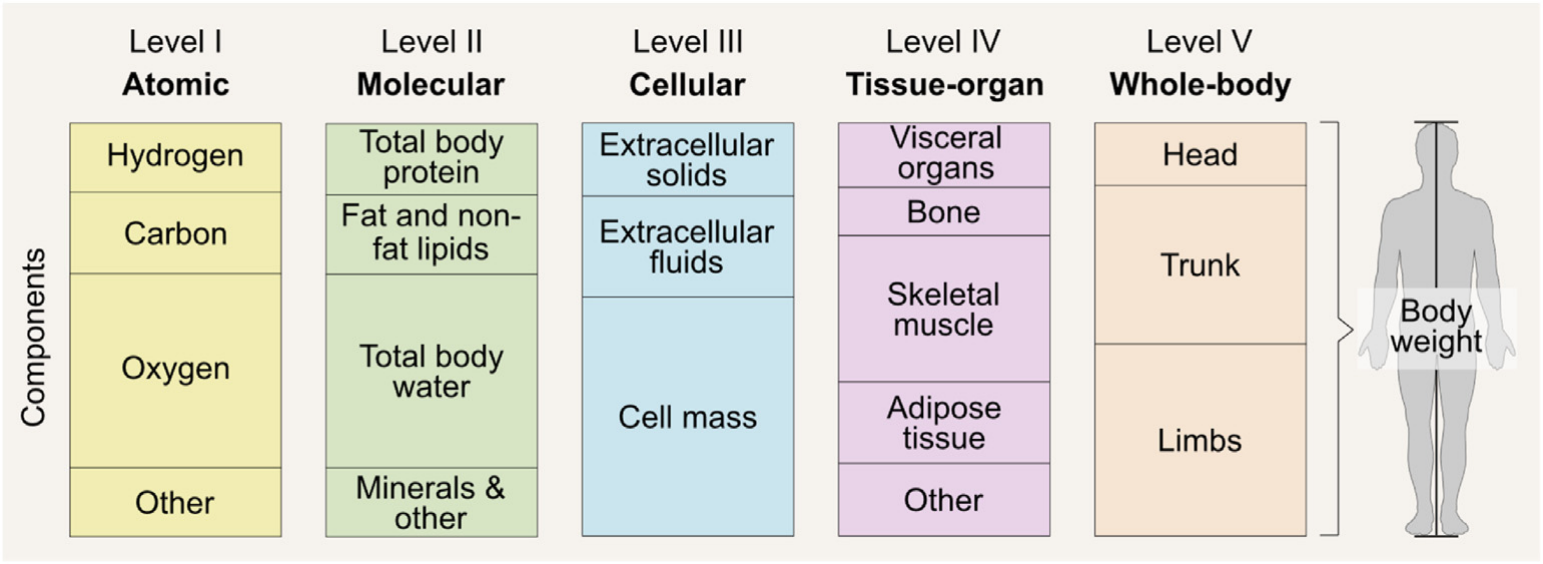
The 5-level body composition organizational framework [3]. Note that fats mainly include nonpolar, or “neutral,” lipids in the form of triglycerides. Nonfat lipids, also termed polar lipids, include phospholipids and glycolipids primarily found in cell membranes [3].

The 5-level organizational framework includes the atomic, molecular, cellular, tissue-organ, and whole-body levels (Figure 2) [3]; total body weight is the combined sum of all components at each of the 5 levels. Models are, therefore, developed at each level and not across levels. Atomic level elements can be measured using in vivo neutron activation analysis and whole-body counting (e.g., with ^40^K), and consist of carbon, hydrogen, oxygen, nitrogen, and other elements present in smaller amounts. This is perhaps the least applied level in body composition assessment, and although important, is not the focus of this review. The molecular level includes water, proteins, lipids, carbohydrates, and other molecules such as the minerals in bone and soft tissues; this is the most used body composition level as many methods are based on its principles. The cellular level includes extracellular fluids (plasma volume, and interstitial fluids), extracellular solids (inorganic and organic), and cell mass. The cell mass component includes body cell mass—the main energy metabolizing component—and fat cells [6,7]. The tissue-organ level of body composition consists of skeletal muscle, adipose tissue (AT), bone, and other tissues and organs (e.g., brain, heart, liver, and spleen). Finally, the whole-body level includes the head, trunk, and limbs. This last level is described by anthropometric measurements such as circumferences, skinfold thicknesses, height, body surface area, and body volume [8]. Each higher level includes all of the lower level components. For example, water at the molecular level consists of hydrogen and oxygen from the atomic level. Because components differ within and between the 5 levels, terminology referring to components assessed at different levels should not be used interchangeably despite their apparent similarity. Fat mass (FM) is assessed at the molecular level and is thus different from AT, which is assessed at the tissue-organ level. Some methods, such as bioelectrical impedance analysis (BIA), may also estimate body components at different levels, depending on the reference method used to develop predictive equations. Parts 2 and 3 of this series will discuss the levels at which each method measures or estimates body components. To understand the methodological differences in body composition research, it is useful to be aware of these variations, as discussed later in this manuscript.

**FIGURE 3:**
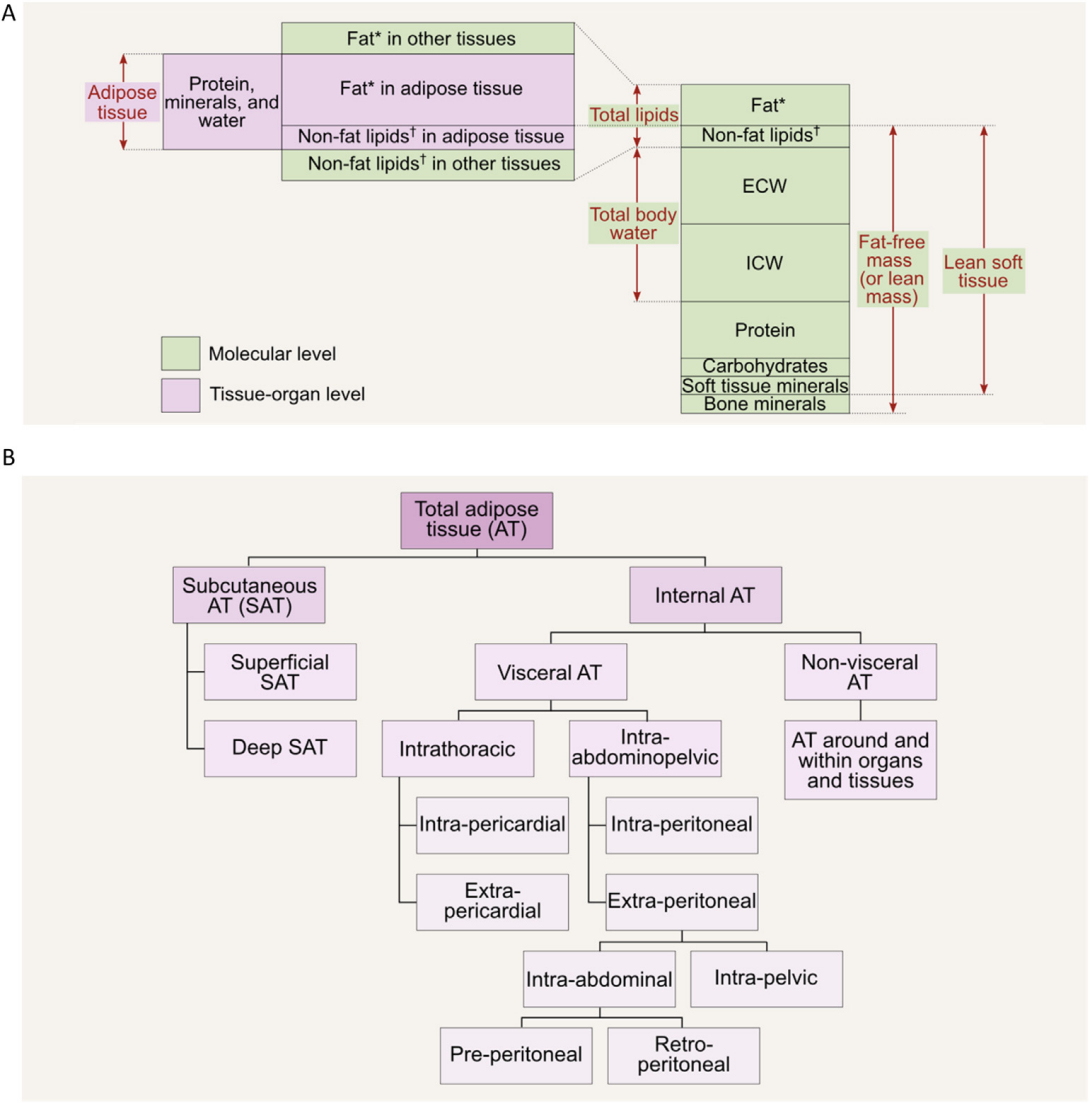
(A) The main components of body composition at the molecular level and how they relate to adipose tissue at the tissue-organ level. Note that protein, mineral, and water are molecular components of adipose tissue, but they are neither fat nor nonfat lipids. Despite being synonymous of fat-free mass, the term “lean mass” should only be used to refer to nonfat or nonadipose tissue components in general, not to a singular body component. (B) Selected subdivisions of adipose tissue [9]. For specific descriptions/definitions and additional adipose tissue classification, refer to the text and Shen et al. [9]. ∗ Nonpolar lipids; ^†^ polar lipids. ^‡^In the lower trunk and gluteal-thigh region, subcutaneous adipose tissue is divided into superficial and deep layers, separated by a fascial plane and differing in morphology and metabolism. AT, adipose tissue; ECW, extracellular water; ICW, intracellular water; SAT, subcutaneous adipose tissue; VAT, visceral adipose tissue.

**FIGURE 4:**
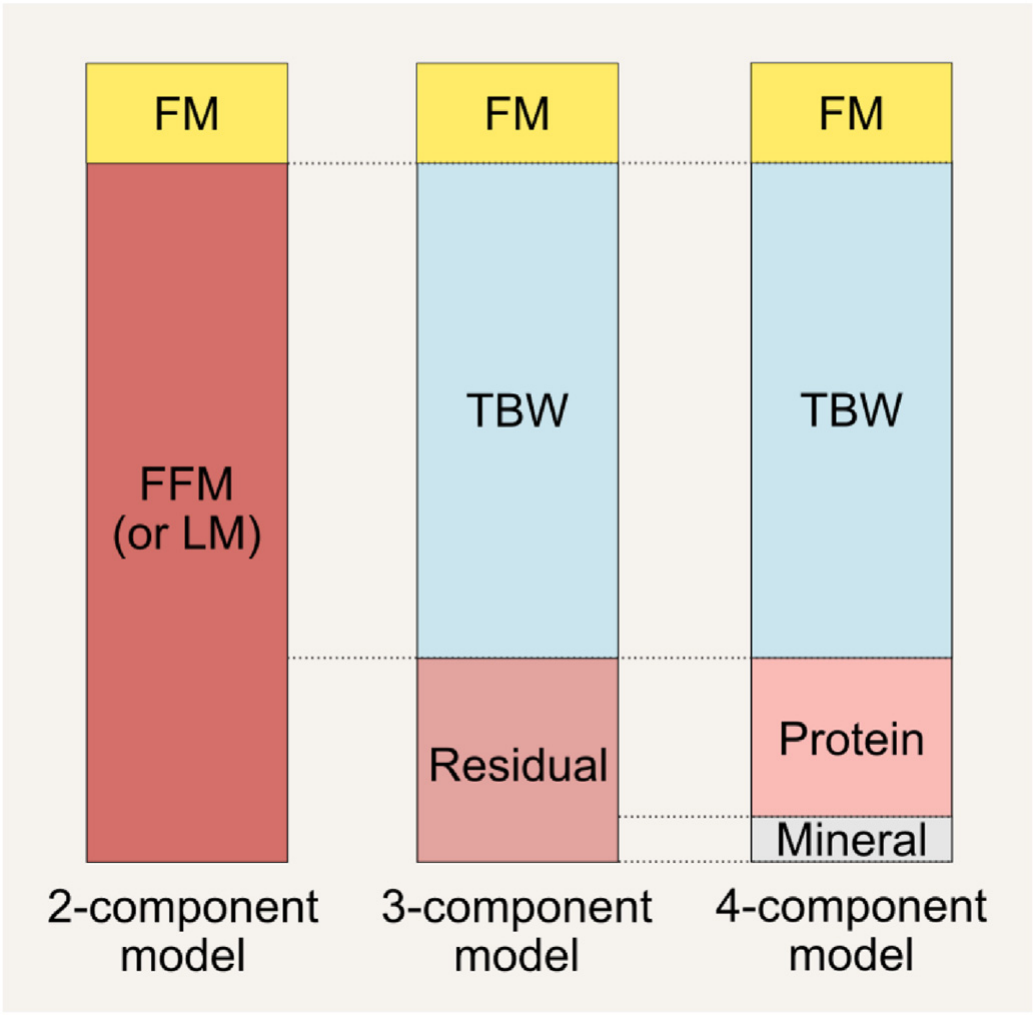
Components of the most used models in body composition assessment. FFM, fat-free mass; FM, fat mass; LM, lean mass; TBW, total body water.

**FIGURE 5:**
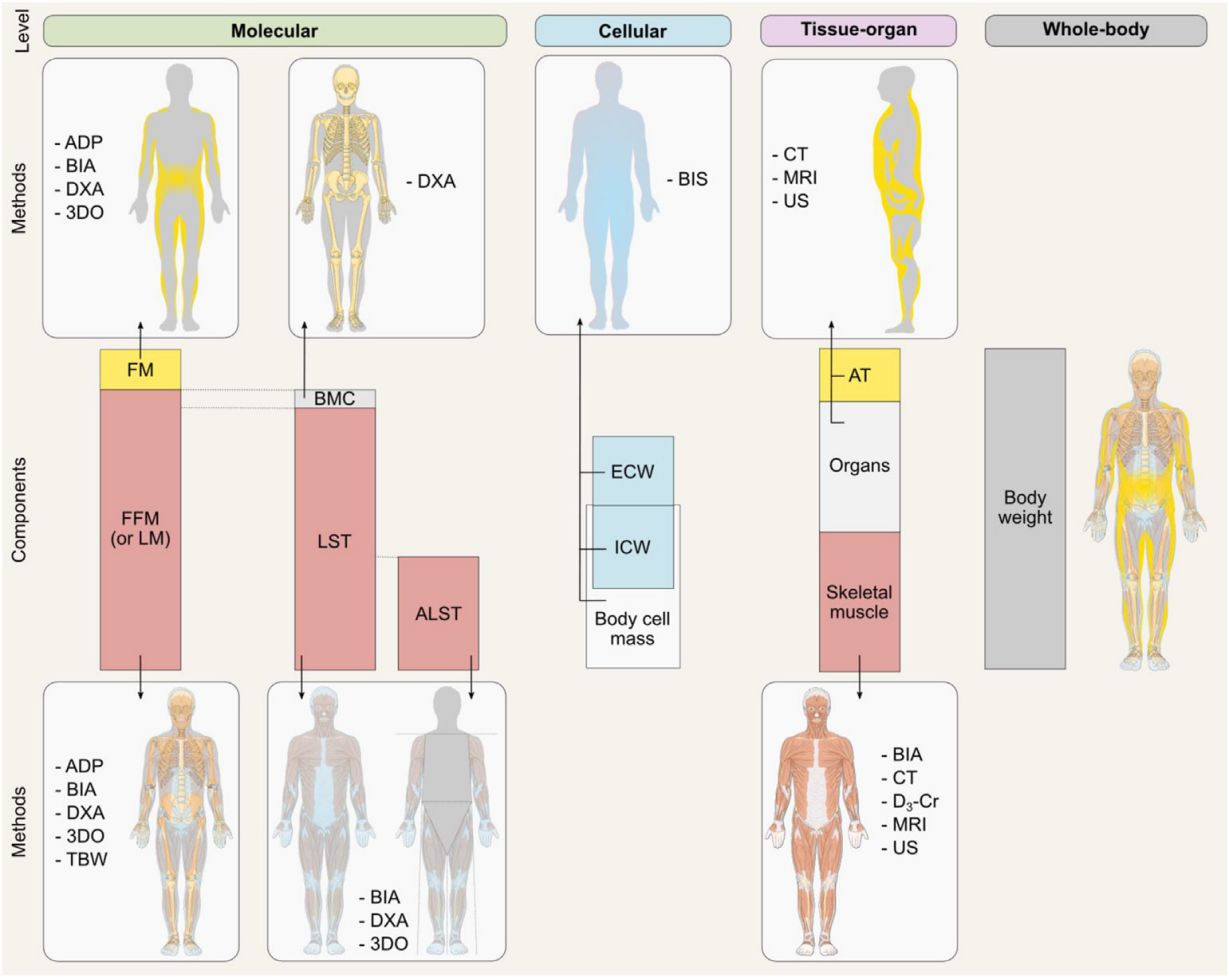
Body components assessed or estimated using common methods at the molecular, cellular, tissue-organ, and whole-body levels. Note that the figure highlights only the most conventionally assessed or estimated body component for each method and level. For example, although computerized tomography is typically used to assess skeletal muscle and adipose tissues, it can also be used to evaluate bone. Furthermore, dual-energy X-ray absorptiometry does not measure individual molecules or molecular groups but does measure a component of fat, which is triglycerides or nonpolar lipids. Images retrieved from smart.servier.com. 3DO, 3D optical; ADP, air displacement plethysmography; ALST, appendicular lean soft tissue; AT, adipose tissue; BIA, bioelectrical impedance analysis; BIS, bioimpedance spectroscopy; BMC, bone mineral content; CT, computerized tomography; D_3_-Cr, D_3_-creatine dilution; DXA, dual-energy X-ray absorptiometry; ECW, extracellular water; FFM, fat-free mass; FM, fat mass; ICW, intracellular water; LST, lean soft tissue; MRI, magnetic resonance imaging; TBW, total body water; US, ultrasound.

By adhering to this 5-level organizational framework, we can effectively measure and understand the complexities of body composition through a range of models. These models enable us to analyze the details within and across each *level* of body composition. Figure 2 [3] categorizes components at each level, yet these categories can be further divided into more specific subdivisions, as demonstrated in Figure 3 [9]. The 2-component model based on FM and fat-free mass (FFM) is the most widely used model at the molecular level. It is applied in methods such as air displacement plethysmography (ADP) and hydrodensitometry, also known as underwater weighing. When 3 or more components are incorporated into the assessment, this is considered a multicomponent body composition model. The 3-component model adds another layer of detail by differentiating body water and residual mass from FFM [10]. The 4-component model includes an additional measurement of minerals, separating the FFM into water, protein, and minerals. Figure 4 summarizes these component models. Multicomponent models go further, with a 6-component model offering the most detailed breakdown, including additional components such as different types of lipid or glycogen stores. Although the 6-component model can be expressed in multiple ways, the chemical model describes body weight in terms of water, protein, bone mineral, cell mineral (i.e., soft tissue minerals), glycogen, and fat [4]. Multicomponent modeling is often considered a reference approach in body composition assessment due to its ability to measure FFM components with minimal assumptions. The reader is referred to in-depth discussion on body composition modeling, which is beyond the scope of this publication [11,12]. Figure 5 and Supplemental Table 1 describe the most conventionally used methods for assessing or estimating body components at various levels. It should be noted that most common body composition methods evaluate the mass and volume of body components, rather than the cellularity (or composition) of these components per se.

## Terminology: Overview and Recommendation Standards

The importance of clear and consistent terminology in body composition research is often underappreciated. As a result, the use of body composition terminology has been variable in the scientific literature, even among experts. This can lead to misunderstandings and challenges in interpreting, comparing, and summarizing (e.g., systematic reviews and meta-analysis) outcomes across various studies without a thorough examination of the methodologies applied and the definition of which component is being assessed. Yet many studies fail to report essential methodological details. Moreover, manufacturers of body composition equipment often use improper terminology, further complicating the issue. It is, therefore, imperative to use accurate terminology when reporting data to understand precisely what each body composition equipment measures or estimates, regardless of the terminology utilized in the equipment’s output.

Recognizing the importance of precision in body composition research, and its relevant clinical applications, we propose and strongly recommend the adoption of standardized terminology and definitions. These standards, described below and summarized in Table 1 [9,[13], [14], [15], [16], [17]] and Figures 2 [3] and 3 [9], account for the specific methods employed in assessing body composition and the specific components being examined. Body composition terminology will be further discussed within the context of each method reviewed in **Parts 2** and **3**. By establishing this guidance, we aim to advance the appropriateness and standardization of body composition terms and definitions to enhance the clarity of research findings. This approach will ensure that studies are accurately interpreted and evaluated in the context of the broader scientific community. Thus, the responsibility of employing accurate terminology lies with clinicians and researchers; interpreting data in accordance with methodological standards such as the proposed herein will be necessary to avoid confusion in the field.

**TABLE 1:** Glossary of body composition terms and suggested abbreviations.

Body composition level	Definition
Molecular level	
Total lipids	Total lipids are carbon- and hydrogen-containing compounds that are relatively insoluble in water and soluble in solvents such as ether, benzene, and alcohol [16]. The complex lipids found in biological systems include polar lipids (also termed as “nonfat lipids”) and nonpolar lipids (also termed as “fat”). Polar lipids consist of phospholipids and glycolipids that function in cell membranes and perform other vital cell functions. Nonpolar or “neutral” lipids are predominantly triglycerides, a long-term energy source [13].
Fat	“Fat” generally refers to esters of fatty acids, or a mixture of such compounds, that occur in living beings. The term refers specifically to triglycerides (triple esters of glycerol) that are the main nonpolar or “neutral” lipids components of fatty tissues in animals [17]. Fat is the major contributor to total body lipids; the remaining lipids are components of the FFM [15].
FFM	Everything in the body, excluding fat (triglycerides). FFM contains nonfat lipids (essential or structural lipids). LBM is not a clearly defined chemical component, and for that reason, early leaders in body composition research agreed to no longer use this term in scientific publications [14]. “Lean mass” is acceptable as a synonym for FFM but should not be confused with LST. Unfortunately, DXA reports incorrectly label LST as lean mass, leading to a common terminological mistake.
LST	LST is the difference between body weight and the sum of fat and BMC, and includes nonfat lipids. LST is a term generally specific to DXA, given DXAs ability to quantify the following 3 components: fat (usually calibrated against triglyceride standards), BMC (the mineral portion of bone), and LST. LST includes 6 regional components: both arms, both legs, trunk, and head. The LST sum of all 4 extremities is referred to as ALST). ALM is the sum of ALST and BMC.
TBW	TBW is a major molecular-level component present in the FFM portion of body mass. TBW is distributed in the ICW and ECW spaces.
BMC	BMC is the mineral portion of bone.
TBP	TBP, found within FFM and LST, includes the intracellular and extracellular proteins across the body’s organs and tissues.
Cellular level	
BCM	This component represents all the body’s cell mass, including ICF and intracellular solids.
ECF	Fluids found in the extracellular space, including ECW, minerals/electrolytes, circulating proteins, and other substrates.
ECS	The remainder of body mass after subtracting BCM and ECF.
Tissue-organ level	
AT	AT is classified as a type of connective tissue composed of adipocytes, collagen, elastic fibers, fibroblasts, capillaries, and ECF. About 80% of AT is made up of storage triglycerides (fat), that are part of the body’s energy reserve. AT includes multiple cell types and is distributed in several subdivisions [9], such as SAT, VAT, and IMAT (single or multiple grouped adipocytes found within skeletal muscles). IntraMAT, is part of IMAT and includes adipocytes within muscle fascicles and accounts for extramyocellular lipids observed with magnetic resonance spectroscopy. Intramyocellular lipids are present within skeletal muscles and consist of an outer phospholipid layer and inner triglyceride core. Intracellular fat is also present in hepatocytes (liver cells).
Skeletal muscle	Skeletal muscle is composed of muscle cells, connective tissues, and other components; when measured in vivo*,* excludes embedded AT and fat cells.

Abbreviations: ALM, appendicular lean mass; ALST, appendicular lean soft tissue; AT, adipose tissue, BCM, body cell mass; BMC, bone mineral content; DXA, dual-energy X-ray absorptiometry; ECF, extracellular fluids; ECS, extracellular solids; ECW, extracellular water; FFM, fat-free mass; ICW, intracellular water; ICF, intracellular fluids; IMAT, intermuscular adipose tissue; intraMAT, intramuscular adipose tissue; LBM, lean body mass; LST, lean soft tissue; SAT, subcutaneous adipose tissue; TBP, total body protein; TBW, total body water; VAT, visceral adipose tissue.

**FIGURE 6:**
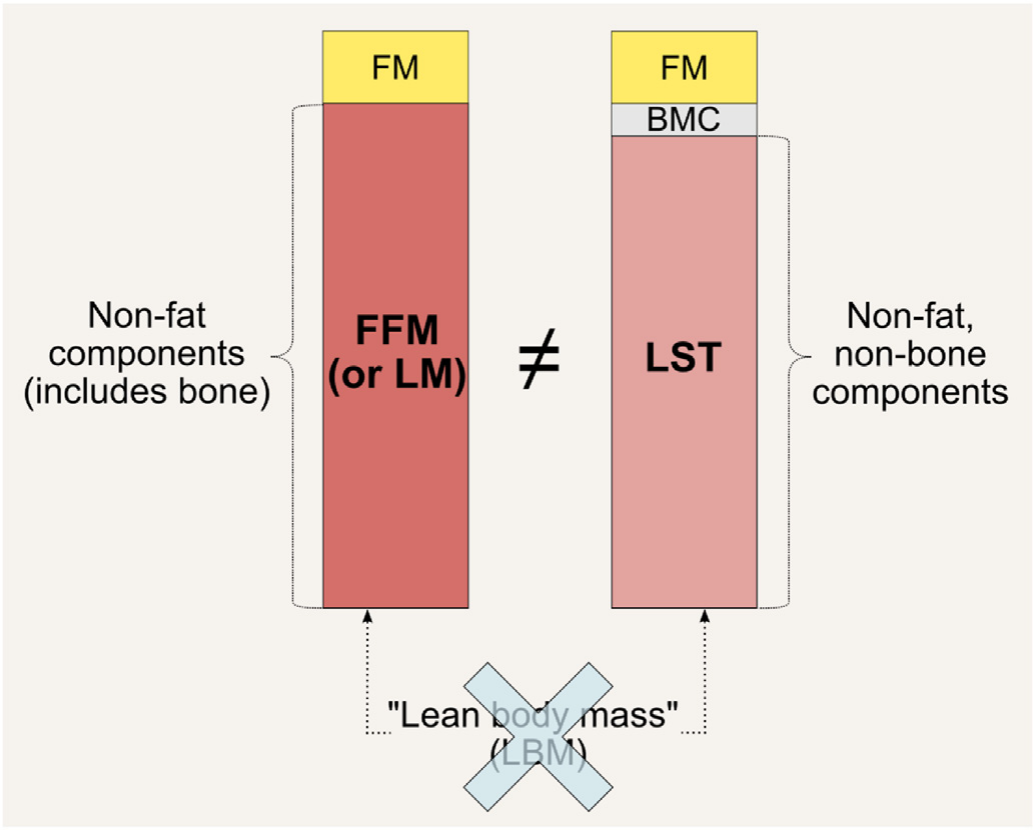
Precise terminology is recommended when describing fat-free mass (FFM) and lean soft tissue (LST), as they represent distinct body components. The term “lean mass” (LM) is synonymous with FFM but not with LST. “Lean mass” may be used as a general reference to nonfat or nonadipose tissue components but not to an actual, singular body component. The term “lean body mass” (LBM) should not be used due to its lack of accuracy [18]. Thus, FFM = LM = LST + bone mineral content (BMC). BMC, bone mineral content; FM, fat mass; FFM, fat-free mass; LBM, lean body mass; LM, lean mass; LST, lean soft tissue.

The term “lean body mass” (LBM) has historically been used ambiguously and inaccurately to denote FFM, lean mass (LM), or lean soft tissue (LST). Initially introduced by Behnke, the term gained widespread use despite its lack of a clear definition [13]. In the 1980s, a consensus among experts established that FFM and LBM were synonymous, with a preference for using FFM due to its more precise definition rooted in chemical composition [14]. Subsequent reports on new body composition models have consistently used the term “FFM.” Given this evolution in understanding, the term “lean body mass” is no longer considered scientifically accurate and should be avoided in favor of more precise terminology (Figure 6) [18]. Nevertheless, it continues to be used by some authors, often out of convention or habit (Supplemental Figure 2). According to this definition, the 2-component molecular-level model is thus: body weight = FM + FFM (Figure 4).

Moving forward, we propose standardized definitions as outlined in Table 1 [9,[13], [14], [15], [16], [17]] and Figures 2 [3] and 3 [9]. “Fat-free mass” is defined as everything in the body excluding fat (mainly neutral, nonpolar lipids in the form of triglycerides). Contrary to what its name suggests, FFM does contain nonfat lipids. A historical explanation of this source of confusion is provided elsewhere [18], whereas the distinction between fat and lipids is discussed below.

**FIGURE 7:**
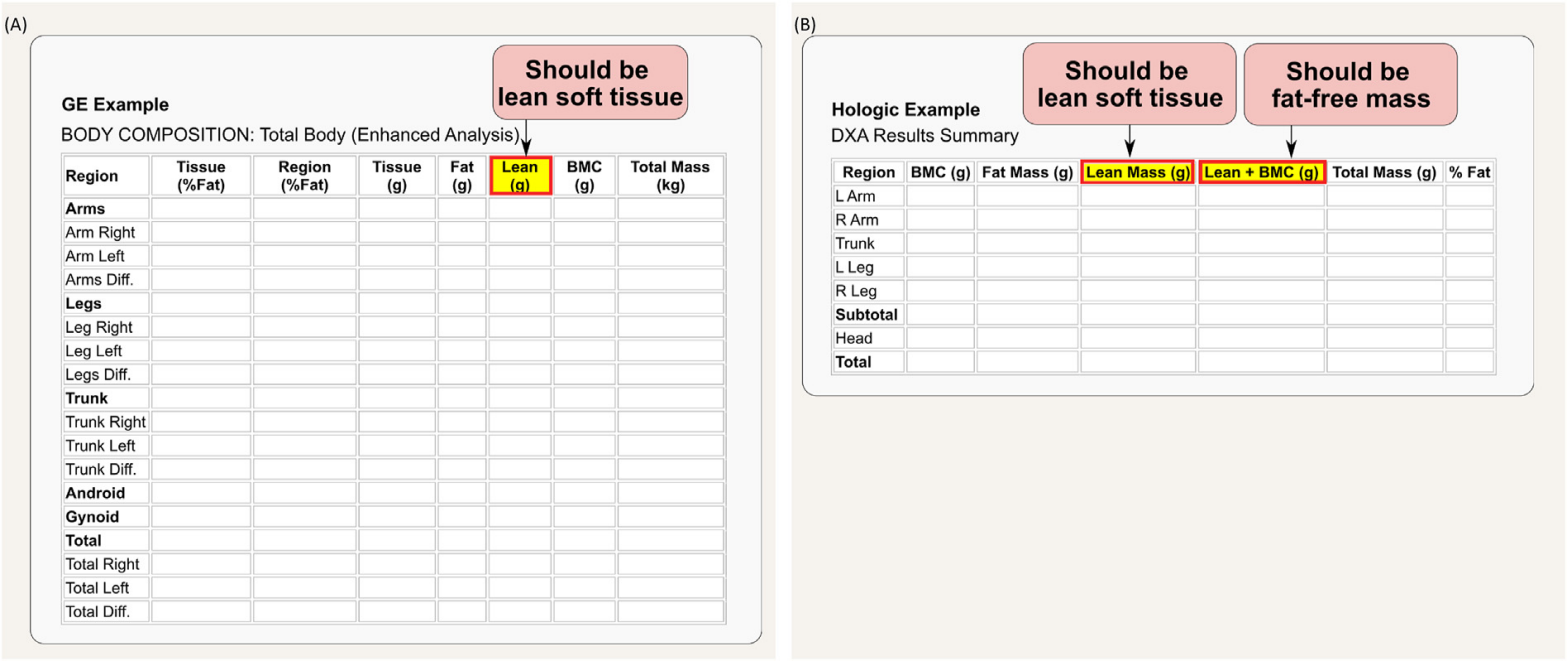
The term “lean mas” is inconsistently defined across dual-energy X-ray (DXA) manufacturers. In the report outputs of 2 manufacturers, “lean” and “lean mass” refer specifically to “lean soft tissue” (A, B), whereas “lean + BMC” represents fat-free mass (B). BMC, bone mineral content.

“Lean mass” is a more convoluted term as it has been loosely used in the literature with many proposed definitions. We do not recommend using this term to refer to an actual, singular body component. We recommend using the term “lean mass” only when generally referring to nonfat or non-AT components (Figure 6) [18]. LM is thus synonymous with FFM but not with LST. “Lean soft tissue,” as the name implies, excludes bone mineral content (BMC), and thus: FFM = LM = LST + BMC. This is important to recognize as the term LM is inconsistently used among dual-energy X-ray absorptiometry (DXA) manufacturers (Figure 7). Finally, the term “skeletal muscle” refers to a tissue-organ level component and not to the 2 molecular-level components FFM and LST. There is also confusion when appendicular LST (ALST) estimated by DXA is incorrectly referred to as “appendicular skeletal muscle,” “appendicular skeletal muscle mass,” or “appendicular lean mass (ALM; the sum of ALST and bone mineral mass)” instead of ALST. This misuse of terminology is common in outputs from BIA devices, as the reference method used to develop the BIA prediction equation ultimately determines which body component is being estimated, an information often omitted by manufacturers. We strongly recommend that manufacturers clearly disclose this information and ensure that access to such critical details is not limited to more expensive models. Similarly, the terminology for the ALST indexed to height-squared should be appendicular lean soft tissue index, rather than appendicular skeletal mass index or appendicular lean mass index (ALMI). ALM indexed to height^2^ is abbreviated as ALMI, corresponding to the FFM presented in the limbs (with bone) adjusted by height,^2^ which is synonymous with appendicular FFM index.

When working with historical datasets or conducting literature reviews that include studies using outdated terminology, researchers should begin by evaluating the body composition method used and how the authors described the body component to determine which specific components were measured. If this information is unclear, it may be necessary to consult original documentation, communicate with corresponding authors, or contact device manufacturers for clarification, especially when dealing with older technologies. We recommend applying the standardized terminology proposed in this manuscript to replace outdated terms in historical datasets and publications wherever appropriate. For example, in NHANES datasets, DXA-derived “lean mass including BMC” and “lean mass excluding BMC” should be updated to FFM and LST, respectively. Additional examples where this approach has been used in literature reviews are provided elsewhere [[19], [20], [21]]. This harmonization will support consistency in reporting and improve comparability across studies, particularly when pooling older and newer datasets.

The distinction between lipids and fat is nuanced. There are several different classification systems for lipids, but a useful one in the current context divides complex lipids into 2 main groups, nonpolar and polar, related to how they are extracted with different solvent systems [[22], [23], [24], [25]]. Nonpolar or “neutral” lipids, which when in solid form are referred to as “fat,” are mainly triglycerides that serve as an energy source when glucose levels drop or when the body requires extra energy. Fat is primarily stored in AT, but it can also be found within intracellular triglyceride pools in muscle, the liver (or other organs), or as circulating lipoproteins. Fats react differently to shifts in energy balance compared with polar lipids (referred to as “nonfat lipids”) [15]. The term “available energy,” when referring to “fat,” typically denotes this storage lipid fraction. Polar or nonfat lipids include phospholipids and glycolipids found primarily in cell membranes; these are included as molecular components in FFM. Behnke termed these “essential” or structural lipids, as they often remain present even in cases of severe starvation [13,26]. Total lipids encompass both “fat” and “nonfat” lipids.

AT contains both nonpolar and polar lipids, the latter found incorporated into adipocyte cell membranes and other intracellular structures. White AT depots are classified based on location: subcutaneous AT is found beneath the skin (dermis); visceral AT is located within the abdominal cavity surrounding internal organs; and intermuscular AT (IMAT) is observed within muscle fibers and between muscle groups [27]. Intramuscular AT (intraMAT) is part of IMAT and includes adipocytes present within muscle fascicles. IntraMAT is detected as extramyocellular lipids with magnetic resonance spectroscopy. IntraMAT is not usually visible on standard computerized tomography (CT) or magnetic ressonance imaging. AT depots—including white and other AT types—may also be located in other regions within the human body, as shown in Figure 3B [9].

Total body water (TBW) is another component of FFM, making up ∼70%–75% of FFM and accounting for 55%–60% of body weight, although both percentages can vary with age, sex, and disease or hydration status. TBW is distributed throughout the body in 2 main compartments: intracellular water (ICW) and extracellular water (ECW). The ICW compartment contains roughly two-thirds of TBW, indicating the substantial volume of water contained within body cells. The remaining one-third is in the ECW, which includes the water in interstitial fluid, plasma, and transcellular fluids.

## Discussion and Perspectives

A clear understanding and differentiation of levels, models, and terminology are crucial for accurate assessment, reporting, and interpretation of body composition data. Here, we clarified these aspects and recommended standardized body composition terminology.

Specifics of common body composition methods will be reported in upcoming publications. The reviewed methods were selected based on their relevance and popularity in both clinical and research use and alignment with what has been proposed in clinical guidelines addressing body composition assessment (e.g., malnutrition, sarcopenia, sarcopenic obesity), as well as what has been proposed as state-of-the-art methods. Upcoming publications will address single- and multiple-frequency BIA, DXA, CT, and ultrasound imaging. We will additionally address ADP, magnetic resonance imaging and spectroscopy, bioimpedance spectroscopy, D_3_-creatine dilution, TBW, and 3D optical scanning in future publications.

## Author contributions

The authors’ responsibilities were as follows – CMP, MCG, KN, SBH: designed and led the group; and all authors: responsible for cowriting the manuscript and reviewed and approved the final manuscript.

## Endorsements

We are seeking endorsements from clinical nutrition societies and affiliated journals to promote consistency and adherence to this terminology.

## Data availability

Data described in the manuscript, code book, and analytic code will not be made available because this is a review article and did not have primary data analysis.

## Funding

CMP is partially supported by the Canada Research Chairs Program. MCG is partially supported by the Brazilian National Council for Scientific and Technological Development (CNPq, Brazil). The funders had no role in the project design, interpretation of evidence, and writing of the manuscript.

## Conflict of interest

CMP has received honoraria and/or paid consultancy from Abbott Nutrition, Nutricia, Nestlé Health Science, AMRA Medical, and Novo Nordisk. MCG reports receiving speaking fees from Abbott Nutrition, Danone/Nutricia, and Nestle Health Science Brazil. TC reports speaking fees from Nutricia, Fresenius-Kabi, Nestle and Abbott. OTC has received research support from Eli Lilly and Co, Nestle, and Novo Nordisk. SBH serves on the Medical Advisory Boards of Tanita Corporation, Novo Nordisk, Lilly, Abbott, Regeneron, and Medifast. KN, RB, CC, GLJ, TA, TGBS, ABW, CPE, WJE, DAF, LG, HHH, MK, JLMC, MM, MJM, CEO, SP, LBS, JAS, MS, BJGS, YY, and SZ have no conflicts of interest.
