# Supplementary material for: Methodological standards for body composition—an expert-endorsed guide for research and clinical applications: levels, models, and terminology
Source: Am J Clin Nutr. 2025 Jul 17;122(2):384–91. doi: 10.1016/j.ajcnut.2025.05.022 (PMC12405783; doi:10.1016/j.ajcnut.2025.05.022)
Supplement: multimedia component 1 [file mmc1.pdf]

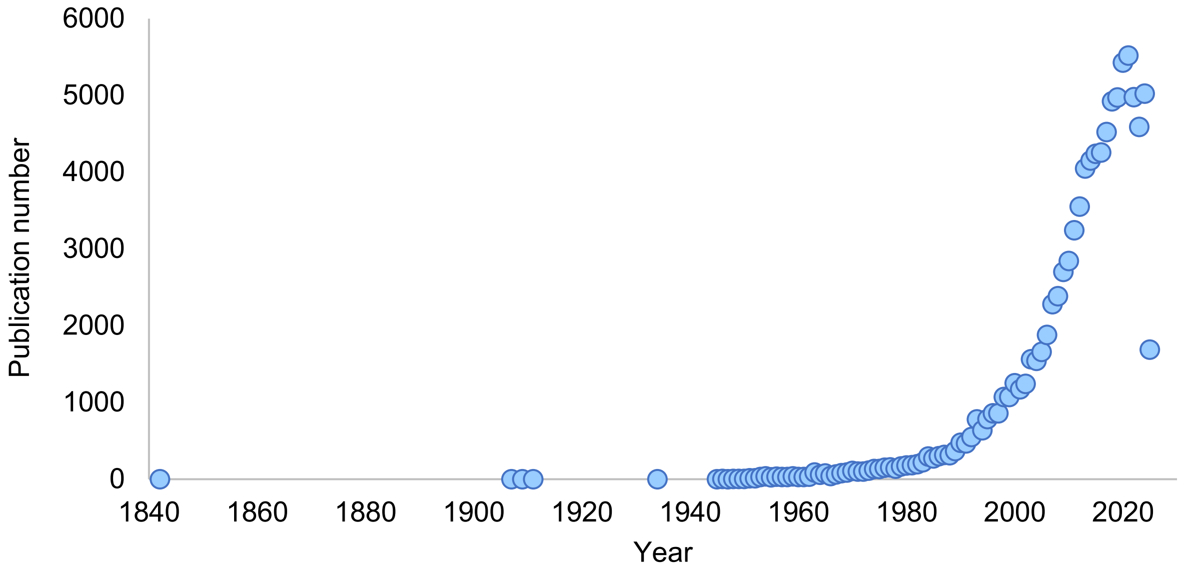

Supplemental Figure 1. Publication rate in human body composition research. The data were obtained through a search conducted on April 24, 2025, using the search string *(human) AND (body composition)* in PubMed.

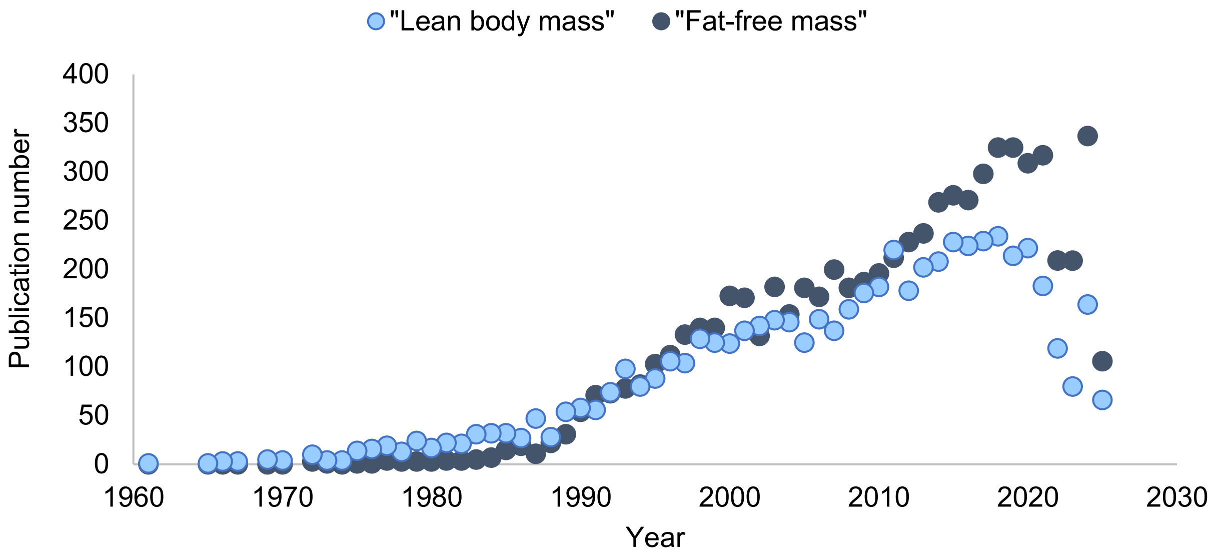

Supplemental Figure 2. Publication trends in human body composition research using the terms “lean body mass” and “fat-free mass”. Data were obtained from a PubMed title/abstract search conducted on April 14, 2025, using the search terms “lean body mass” and “fat-free mass” in separate queries, with filters applied for humans and adults (age ≥19 years).

Level	Components	Methods
Level I – Atomic	TBK	Whole-body K counting
	Sodium, chlorine, phosphorus, and calcium	Delayed-γ neutron activation
	Nitrogen	Prompt-γ neutron activation
	Carbon	Inelastic neutron scattering
Level II – Molecular	FM, FFM (or LM)	ADP, BIA, DXA, 3DO
	LST, ALST	BIA, DXA, 3DO
	BMC	DXA
	TBW	Isotope dilution methods
Level III – Cellular	ECF[1], ICF[1], BCM	BIS, isotope dilution methods
Level IV – Tissue-organ	AT, organs	CT, MRI, US
	Skeletal muscle	CT, MRI, US, BIA, D_3-Cr
Level V – Whole-body	Body weight, volume, stature, circumferences, skinfold thickness	Anthropometry

Supplemental Table 1. Body components assessed or estimated using select methods.

[1]Note that when measuring these spaces, they are most often represented as intracellular water and extracellular water versus intracellular fluid and extracellular fluid, respectively. 3DO: 3D optical, ADP: air displacement plethysmography, ALST: appendicular lean soft tissue, AT: adipose tissue, BIA: bioelectrical impedance analysis, BIS: bioimpedance spectroscopy, BMC: bone mineral content; CT: computerized tomography, D_3-Cr: D_3-creatine dilution, DXA: dual-energy X-ray absorptiometry, ECF, extracellular fluid, FFM: fat-free mass, FM: fat mass, ICF, intracellular fluid, LM, lean mass; LST: lean soft tissue, MRI: magnetic resonance imaging, TBW: total body water, TBK, total body potassium, US: ultrasound
